# Co-evolution of Enzymes Involved in Plant Cell Wall Metabolism in the Grasses

**DOI:** 10.3389/fpls.2019.01009

**Published:** 2019-08-09

**Authors:** Vincent Bulone, Julian G. Schwerdt, Geoffrey B. Fincher

**Affiliations:** ^1^Australian Research Council Centre of Excellence in Plant Cell Walls, School of Agriculture, Food and Wine, University of Adelaid, Glen Osmond, SA, Australia; ^2^Adelaide Glycomics, School of Agriculture, Food and Wine, University of Adelaide, Glen Osmond, SA, Australia

**Keywords:** cereals, (1,3;1,4)-β-glucans, grasses, heteroxylans, plant cell walls, polysaccharide hydrolases, polysaccharide synthases

## Abstract

There has been a dramatic evolutionary shift in the polysaccharide composition of cell walls in the grasses, with increases in arabinoxylans and (1,3;1,4)-β-glucans and decreases in pectic polysaccharides, mannans, and xyloglucans, compared with other angiosperms. Several enzymes are involved in the biosynthesis of arabinoxylans, but the overall process is not yet defined and whether their increased abundance in grasses results from active or reactive evolutionary forces is not clear. Phylogenetic analyses reveal that multiple independent evolution of genes encoding (1,3;1,4)-β-glucan synthases has probably occurred within the large cellulose synthase/cellulose synthase-like (CesA/Csl) gene family of angiosperms. The (1,3;1,4)-β-glucan synthases appear to be capable of inserting both (1,3)- and (1,4)-β-linkages in the elongating polysaccharide chain, although the precise mechanism through which this is achieved remains unclear. Nevertheless, these enzymes probably evolved from synthases that originally synthesized only (1,4)-β-linkages. Initially, (1,3;1,4)-β-glucans could be turned over through preexisting cellulases, but as the need for specific hydrolysis was required, the grasses evolved specific (1,3;1,4)-β-glucan endohydrolases. The corresponding genes evolved from genes for the more widely distributed (1,3)-β-glucan endohydrolases. Why the subgroups of CesA/Csl genes that mediate the synthesis of (1,3;1,4)-β-glucans have been retained by the highly successful grasses but by few other angiosperms or lower plants represents an intriguing biological question. In this review, we address this important aspect of cell wall polysaccharide evolution in the grasses, with a particular focus on the enzymes involved in noncellulosic polysaccharide biosynthesis, hydrolysis, and modification.

## Introduction

Grasses of the Poaceae family are arguably the most successful land plants on the planet. They are estimated to cover 20% of the surface of Earth, from Antarctica to the equator and from sea level to our highest mountains (Gaut, 2002). The grasses evolved relatively recently, probably between 70 and 55 million years ago (Kellogg, 2001). The ecological dominance of the grasses is mainly attributable to the co-evolution of their basal meristem with the evolution of large and diverse native herbivores (Stebbins, 1981). It has been suggested that the basal meristem offers quicker recovery from grazing or fire damage than almost all nongrass plant species (Stebbins, 1981; Brooks et al., 2004). Thus, grasses are important components of fodder and forage for both native herbivores and domestic livestock.

In addition to their ecological dominance, the grasses assume unparalleled economic importance through the role of major species such as corn, wheat, rice, barley, millet, oats, and sugarcane in the provision of the major proportion of daily caloric intake for most human societies (Kellogg, 2001). The domestication of wild, ancestral relatives of these cereal species in the Fertile Crescent between 12,000 and 9,500 years ago enabled humans to advance from hunter-gatherers to agrarian farmers (Harlan and Zohary, 1966; Willcox, 2013). Domestication of the cereals involved a number of significant changes in morphology, including an increase in grain size and the evolution of nonbrittle rachises (Purugganan and Fuller, 2009). A pronounced thickening of cell walls is associated with the nonbrittle rachis phenotype, but precisely how this might affect brittleness in the disarticulation zone is not known (Pourkheirandish et al., 2015). Large-scale production of the domesticated cereal crop species not only allowed early farmers to provide basic foods for larger social groups, but it also enabled those groups to collect sufficient grain for brewing alcoholic beverages. Indeed, it is likely that human demands for both food and beer, coupled with farmer intervention through their selection activities, were major driving forces in the evolution and development of crop species for human societies (Hayden, 2003).

The cell walls of cereals and other grasses have characteristics that have proved important in practical applications of cereal species beyond bread making and brewing. For example, soluble dietary fiber from the cell walls of cereal products has been recognized as a crucial component for human health and nutrition (Bingham et al., 2003; Jacobs and Gallaher, 2004; Jemal et al., 2005; Collins et al., 2010). In addition, lignocellulosic residues of cell walls from cereal straw and bran have attracted considerable attention for the production of renewable liquid biofuels (Burton and Fincher, 2014a; Loqué et al., 2015; McCann and Carpita, 2015; Fry, 2017; Biswal et al., 2018) and of other biomaterials with valuable commercial applications (Dhugga, 2007; Bar-Cohen, 2012). A key distinguishing characteristic of the grasses is the composition of their cell walls, which exhibit dramatic evolutionary changes compared with the walls of other angiosperm species. In this short review, we examine the evolution of the cell wall components of the grasses, with a special emphasis on the evolution of the synthase enzymes that are responsible for the biosynthesis of matrix phase (1,3;1,4)-β-glucan polysaccharides, together with the co-evolution of hydrolytic enzymes that specifically degrade these (1,3;1,4)-β-glucans. In addition, we briefly examine the evolution of enzymes involved in heteroxylan biosynthesis.

## A Significant Evolutionary Shift Occurred in Cell Walls of the Grasses

In general terms, the cell walls of the angiosperms consist of cellulosic microfibrils embedded in a matrix phase of noncellulosic polysaccharides and lignin, with up to 10% of wall-bound protein (McCann and Knox, 2018). The cellulose microfibrils provide the wall with high levels of tensile strength, while the noncellulosic matrix phase polysaccharides and, in particular, lignin provide the compressive strength and the resistance to shear forces required for wall function (Kerstens et al., 2001). The wall can be further strengthened through extensive thickening, as seen in secondary walls, and through lamination of layers with differing microfibril orientation within the secondary walls.

**Figure 1:**
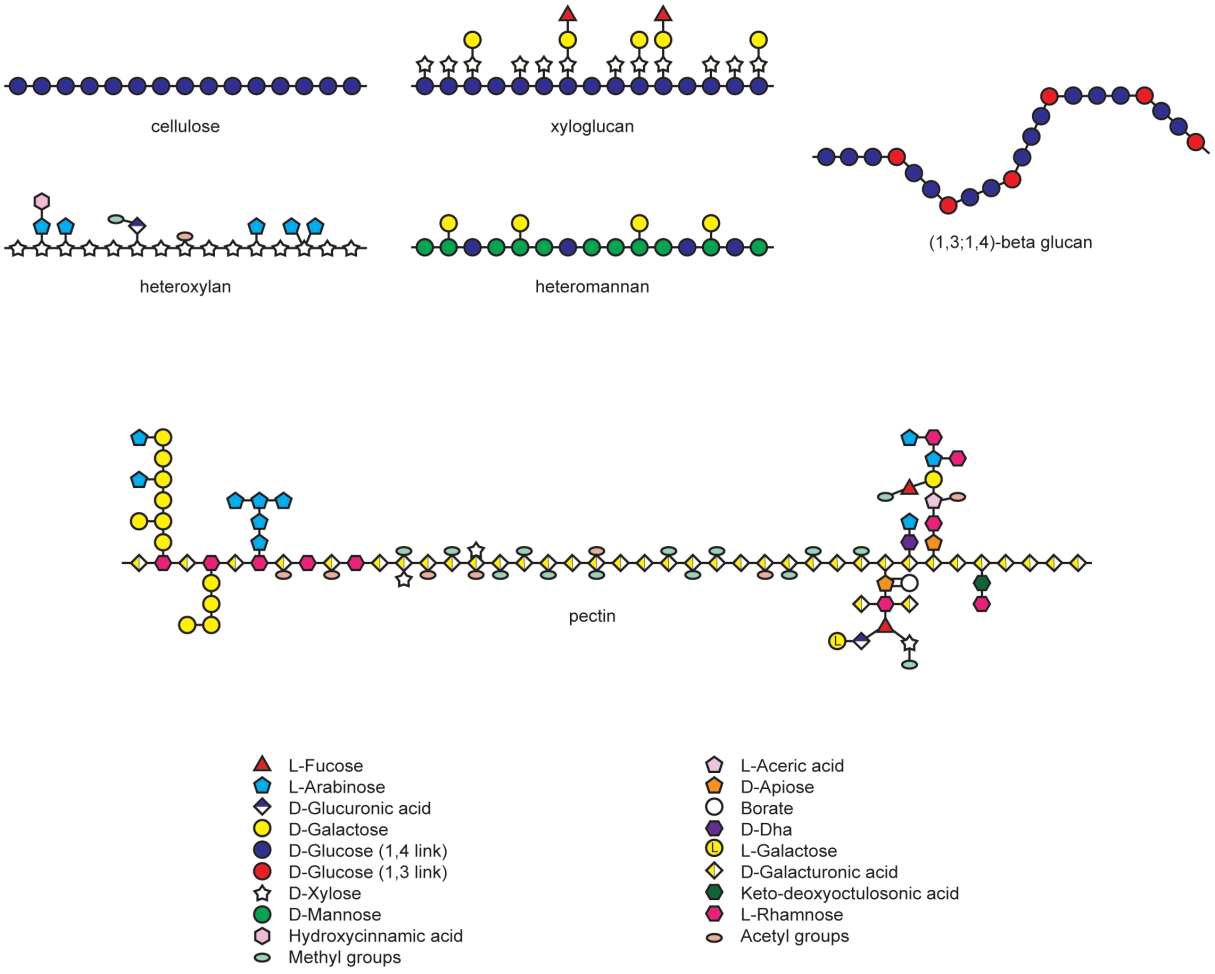
Heterogeneity in structures of wall polysaccharides in angiosperms. Structures redrawn from Mohnen (2008), Burton et al. (2010), and Doblin et al. (2010).

The noncellulosic polysaccharides of the matrix phase of walls in angiosperms are highly diverse in their chemistry but closely homologous with respect to their physiochemical properties (Figure 1; Popper et al., 2011). The structures of xyloglucans, heteroxylans, and heteromannans are based on an essentially linear backbone of (1,4)-β-linked monosaccharides (Scheller and Ulvskov, 2010), while the backbone of galacturonans, which are major constituents of pectic polysaccharides, are comprised of (1,4)-α-linked chains of galacturonosyl residues (Figure 1; Mohnen, 2008). Chain aggregation of the type found in cellulose microfibrils is prevented in the noncellulosic wall polysaccharides by the substitution of the backbone chain with short oligosaccharides, monosaccharides, or acetyl groups. This is exemplified by the xyloglucans of dicot walls, which consist of a (1,4)-β-glucan backbone substituted with xylosyl residues and short oligosaccharides. Similarly, the heteroxylans that are abundant in the walls of grasses have a linear (1,4)-β-xylan backbone, which is similar in overall conformation to a cellulose molecule but which is substituted with arabinosyl and other mono- or oligosaccharides to limit aggregation (Figure 1). The major exception to this strategy is found in the (1,3;1,4)-β-glucans, where main chain aggregation is hindered by the irregular insertion of (1,3)-β-linkages, which cause irregularly spaced molecular kinks and hence an irregular conformation in the unsubstituted polysaccharide backbone that is otherwise composed of linear “cellulosic” (1,4)-β-glucosyl residues (Figure 1).

The evolutionary shift that occurred during the evolution of the grasses resulted in a marked decrease in the abundance of pectic polysaccharides, heteromannans, and xyloglucans in the grasses, with concomitant increases in the abundance of heteroxylans. In addition, walls of the grasses often contain substantial levels of (1,3;1,4)-β-glucans, which are not widely distributed in other plant species (Burton et al., 2010). Although these polysaccharides have diverse chemistries, their physicochemical and solution properties are similar and it is therefore not clear whether the differences reflect any significant mechanical changes in the wall. However, as mentioned below, the (1,3;1,4)-β-glucans might also perform a storage function. These differences in polysaccharide compositions are apparent in Table 1, where selected examples of walls from various tissues of grasses and dicotyledonous angiosperms are compared (Burton and Fincher, 2014b). In a recent study, Okekeogbu et al. (2019) proposed that the distinctive compositions of grass and nongrass cell walls is a result of differential gating or metabolism of secreted polysaccharides from the Golgi apparatus and therefore does not necessarily result from the differential expression of genes encoding specific polysaccharide synthases.

**Table 1:** Comparison of wall composition in tissues of the grasses and dicots.

Tissue	Hetero-xylan	(1,3;1,4)-β-glucan	Cellulose	Hetero-mannan	Pectin	Xyloglucan	References
Barley coleoptiles (4 days)	32	10	35	nr	12	10	(Gibeaut et al., 2005)
Barley aleurone	71	26	2	2	nd	nd	(Bacic and Stone, 1981)
Barley starchy endosperm	20	70	3	2	nd	nd	(Fincher, 1975)
Maize internodes	46	3	35	2	trace	6	(Zhang et al., 2014)
Brachypodium whole grain	4.7	42.4	6	trace	nr	nr	(Guillon et al., 2011)
Rice endosperm	32	nr	36.3	nr	7.3	nr	(Shibuya and Iwasaki, 1985)
*Arabidopsis* leaves	4	0	14	nr	42	20	(Zablackis et al., 1995)
Grape berries (114 dpa)	7	0	31	3	45	8	(Nunan et al., 1998)

*nd, not detected; nr< not reported. Data from Burton and Fincher (2014b)*.

## Fine Structure of (1,3;1,4)-β-Glucans

The (1,3;1,4)-β-glucans of grass cell walls are characterized by both random and nonrandom structural features. Overall, the polysaccharides contain about 70% (1,4)-β-glucosyl residues and 30% (1,3)-β-glucosyl residues, although these values are quite variable. It has been shown in several (1,3;1,4)-β-glucans that most of the polymer consists of β-cellotriosyl and β-cellotetraosyl residues linked by single (1,3)-β-linkages, as follows:

**(non-red)……G3G4G4G3G4G4G3G4G4G3G4G4G4G3G4G4G3G4G4G4G3…..(red),**

where G denotes a β-glucosyl residue, 3 and 4 denote linkage positions, and the nonreducing and reducing ends of the polysaccharide are indicated. The β-cellotriosyl and β-cellotetraosyl residues are underlined. The nonrandom component of (1,3;1,4)-β-glucan structure is represented by the single (1,3)-β-glucosyl residues; two or more contiguous (1,3)-β-glucosyl residues are seldom if ever reported. Given the presence of up to 30% or more (1,3)-β-glucosyl residues in the polysaccharide, random arrangement of these linkage types would clearly result in many instances where two or more adjacent (1,3)-β-glucosyl residues would be found. In addition, it has been shown that the presence of just two contiguous (1,3)-β-glucosyl residues in one (1,3;1,4)-β-glucan molecule is incompatible with the overall three-dimensional structure of the polysaccharide (Buliga et al., 1986).

The random component of (1,3;1,4)-β-glucan structure is represented by the arrangement of the (1,3)-linked β-cellotriosyl and β-cellotetraosyl residues. Markov chain analysis of the sequence of β-cellotriosyl and β-cellotetraosyl residues in barley (1,3;1,4)-β-glucans showed that these two oligosaccharides were randomly distributed (Staudte et al., 1983). The random distribution of these oligosaccharides ensures that the molecular “kinks” caused by the (1,3)-β-glucosyl linkages are also arranged randomly along the chain. This, in turn, limits the ability of the polysaccharide chains to align over long distances to form insoluble aggregates. The ability of the cell to alter the ratio of the β-cellotriosyl and β-cellotetraosyl residues provides a mechanism whereby the solubility of the polysaccharide can be fine-tuned and tailored to the local biological requirements, as described in more detail below. Most (1,3;1,4)-β-glucans also contain longer blocks of up to 12 adjacent (1,4)-β-glucosyl residues (Woodward et al., 1983), but the arrangement of these within the polysaccharide chain is as yet undefined.

As noted above, the relative amounts of (1,3)- and (1,4)-β-glucosyl residues vary in (1,3;1,4)-β-glucans from different sources, and this results in different amounts of β-cellotriosyl and β-cellotetraosyl residues. The fine structure of (1,3;1,4)-β-glucans is often expressed as a ratio of the two oligosaccharides, which can be easily defined with (1,3;1,4)-β-glucan endohydrolases that specifically hydrolyse (1,4)-β-glucosyl linkages on the reducing end side of the (1,3)-β-glucosyl residues in the polysaccharide. The degradation products are easily separated and ratios of trisaccharides and tetrasaccharides released are expressed as the DP3:DP4 ratio, where DP denotes the degree of polymerization of the oligosaccharides (Lazaridou and Biliaderis, 2007; Little et al., 2018). The DP3:DP4 ratio also serves as a useful predictor of the solubility of a particular (1,3;1,4)-β-glucan, where very high or very low ratios indicate that the polysaccharide will exhibit low solubility, while ratios around 1.0:1–2.5:1 are associated with (1,3;1,4)-β-glucans of relatively higher solubility in aqueous media (Lazaridou and Biliaderis, 2007; Burton et al., 2010). Very-high or very-low DP3:DP4 ratios reflect high levels of β-cellotriosyl and β-cellotetraosyl residues, respectively, and in both cases, the (1,3)-β-glucosyl linkages become more evenly spaced. As a result, adjacent polysaccharide chains can align over increasing distances and their solubility in aqueous media will decrease concomitantly. Examples of different (1,3;1,4)-β-glucans that illustrate these characteristics are shown in Table 2.

**Table 2:** Effects of (1,3;1,4)-β-glucan fine structure on solubility.

Species	Cellotriosyl:cellotetraosyl ratio	Solubility
*Equisetum fluviatile* (horsetail fern)	0.1:1.0	Low solubility (mostly cellotetraosyl units)
*Avena sativa* (oats)	1.5:1.0 to 2.3:1.0	Ratio in grain approx. 2.0:1.0 Approx. 80% soluble in H_2_O at 40^°^C Comprises 3.8–6.1% (w/w) of grain
*Hordeum vulgare* (barley)	1.8:1.0 to 3.5:1.0	Ratio in grain approx. 2.5:1.0 Approx. 20% soluble in H_2_O at 40^°^C Comprises 2–10% (w/w) of grain
*Triticum aestivum* (wheat)	3.0:1 to 4.5:1.0	Ratio in grain approx. 2.8:1.0 Essentially insoluble in H_2_O at 40^°^C Comprises 0.5–2.3% (w/w) of grain
*Cetraria islandica* (Icelandic moss)	20.2:1 to 24.6:1.0	Low solubility (mostly cellotriosyl units)
*Rhynchosporium secalis* (barley scald fungus)	More than 10.0:1.0	Low solubility (mostly cellotriosyl units)

## Evolution of Genes Coding for Enzymes That Catalyze (1,3;1,4)-β-Glucan Synthesis

**Figure 2:**
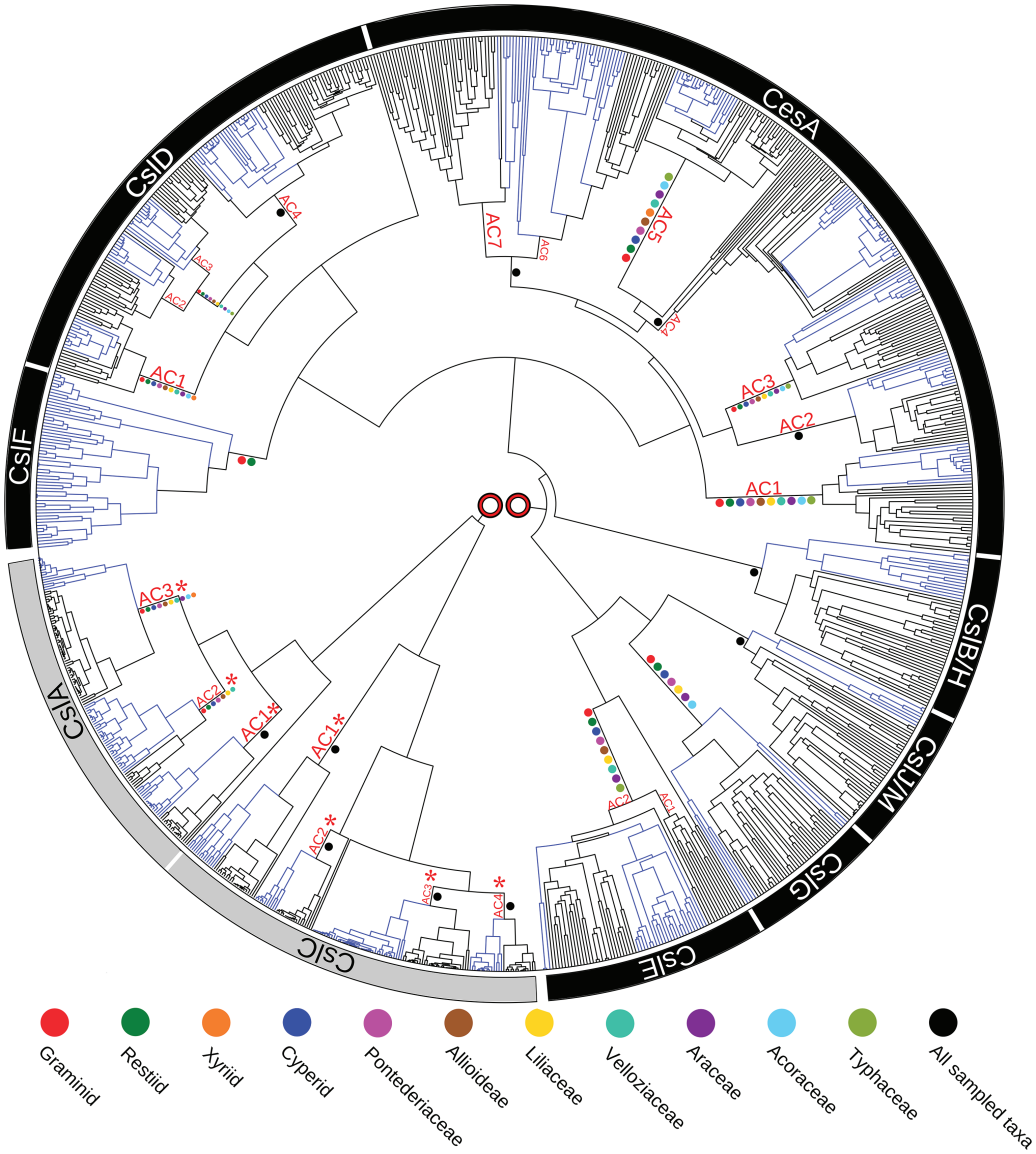
Phylogenetic tree of the cellulose synthase gene superfamily constructed from species with fully sequenced genomes. Blue colored branches indicate monocots, while black branches indicate eudicots. Ancestral Clusters (AC) represent clades that existed before the monocot-eudicot divergence. Colored dots on nodes are from transcriptome data. No direct connection is shown between the two major evolutionary groupings (*CslA/CslC* and *CslF/CslA/CesA/CslB/H/CslJ/M/ CslG/CslE*), because it is likely that the family of genes originated from two separate endosymbiotic events. This figure is reproduced from Little et al. (2018).

Enzymes that direct the biosynthesis of (1,3;1,4)-β-glucans were initially identified through molecular genetics approaches. Given that these polysaccharides have at least some structural similarities to the (1,4)-β-glucan, cellulose, it seemed likely that the genes encoding the synthases might be found in the large *cellulose synthase* (*CesA*) gene family. Indeed, three groups from within the *CesA* superfamily that were believed to be specific for the Poaceae have been implicated in (1,3;1,4)-β-glucan synthesis. These include the *cellulose synthase-like* genes *CslF*, *CslH* (Richmond and Somerville, 2000; Hazen et al., 2002) and *CslJ* (Farrokhi et al., 2006). A recently revised phylogeny of the *CesA* gene family in angiosperms revealed that the three *CslF*, *CslH*, an*d CslJ* clades of the gene superfamily independently co-evolved in the *CesA/CslD/CslF* group, the *CslB/CslH* group and the *CslE/CslG/CslJ/CslM* group, respectively (Figure 2; Little et al., 2018). Similar results have been reported specifically for wheat (Kaur et al., 2017). Current evidence suggests that the *CslF* group evolved from the *CslD* subfamily (Little et al., 2018), which has been implicated in wall biosynthesis in root hairs and pollen tubes, where the encoded enzymes possibly synthesize single, noncrystalline chains of cellulose (Doblin et al., 2001; Kim et al., 2007; Bernal et al., 2008). However, there is also a report that one *CslD* gene encodes a mannan synthase (Yin et al., 2011). The *CslH* clade is the monocot-specific sister clade to the eudicot-specific *CslB*, and probably arose *via* gene duplication, while the *CslJ* clade is the monocot-specific sister to the larger *CslM* clade of the eudicots (Figure 2; Little et al., 2018). Members of the *CslF*, *CslH*, and *CslJ* clades have all been shown to mediate (1,3;1,4)-β-glucan synthesis in heterologous expression systems (Burton et al., 2006; Doblin et al., 2009; Little et al., 2018), although it is not clear if every gene in these groups directs (1,3;1,4)-β-glucan synthesis *in vivo*.

These analyses indicate that plants independently co-evolved the capability for (1,3;1,4)-β-glucan synthesis at least three times through convergent evolution or, alternatively, that the synthesis of this polysaccharide was gained and lost several times during the evolution of the *CesA* gene family of the monocots (Little et al., 2018). Examination of selection pressure exerted on these genes showed that significant positive selection has occurred on the *CslF7* gene and that several amino acid residues in the CslF6 enzyme are also under positive selection pressure (Schwerdt et al., 2015).

## Distribution of (1,3;1,4)-β-Glucans

It was initially believed that the distribution of (1,3;1,4)-β-glucans was largely limited to the Poaceae family in the Order Poales. Related forms of the polysaccharide were later reported in the monilophyte genus *Equisetum* (Fry et al., 2008; Sørensen et al., 2008), bryophytes (Popper and Fry, 2003), certain green and red algae (Lechat et al., 2000), lichens (Honegger and Haisch, 2001; Carbonero et al., 2005), and the fungus *Rhynchosporium secalis* (Harris and Fincher, 2009; Pettolino et al., 2009). Using data from genome sequences, transcript profiles, biochemical analyses, and immunocytochemistry, Little et al. (2018) confirmed that (1,3;1,4)-β-glucans are not restricted to the Poaceae.

**Figure 3:**
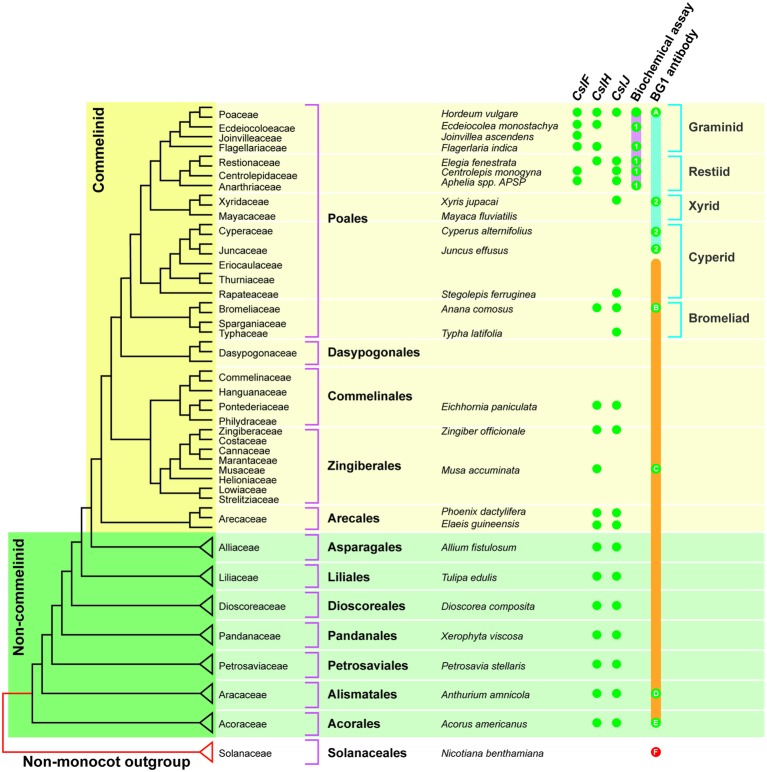
Distribution of (1,3;1,4)-β-glucans in commelinid and noncommelinid species of the monocots. The figure is reproduced from Little et al. (2018) and is based on a species tree adapted from McKain et al. (2016). The orange bar represents species where one would expect (1,3;1,4)-β-glucan antibody labeling based on the presence of *CslH* or *CslJ*. Green dots indicate confirmed antibody labeling; red dots are negative. The pink bar shows species that are predicted to contain (1,3;1,4)-β-glucan as detected with the biochemical assay; actual positives are highlighted using green dots. Green dots labeled 1 are the results of Smith and Harris (1999). The solid blue bar indicates species that are predicted to contain (1,3;1,4)-β-glucan detectable with the BG1 antibody, with actual positives highlighted with green dots. Green dots labeled 2 indicate positive results from Trethewey et al. (2005). The solid orange bar indicates the range of species that are predicted to contain (1,3;1,4)-β-glucan detectable with the BG1 antibody, with positives highlighted using green dots. It should be noted that the Poaceae family lies within the graminid clade. Reproduced from Little et al. (2018).

The *CslF* genes are found in the commelinid monocots, while the *CslH* and *CslJ* genes are broadly distributed across both commelinid and noncommelinid monocots (Figure 3). Biochemical analyses have detected (1,3;1,4)-β-glucan in the graminids and restiids, while the more sensitive immunocytochemical assay also detected this polysaccharide in the xyrids, cyperids, and bromeliads. In addition, it appears that (1,3;1,4)-β-glucans are present in the distantly related commelinid *Musa acuminata* (banana) and in two species from the distantly related noncommelinids, namely *Acorus americanus* and *Anthurium amnicola* (Figure 3). The Poaceae family lies within the graminid group (McKain et al., 2016). In summary, it has become clear that (1,3;1,4)-β-glucans are not restricted to the Poaceae but can be detected in both commelinid and noncommelinid monocots (Figure 3), although they are most broadly found in the Poaceae and found only sporadically in other species.

## Are (1,3;1,4)-β-Glucans Used as a Secondary Storage of Metabolizable Glucose?

The wide retention of (1,3;1,4)-β-glucans in the Poaceae raises the question as to whether there is any competitive advantage conferred on the grasses by these polysaccharides. Firstly, it is important to note that (1,3;1,4)-β-glucans do not appear to be an essential component of walls in the grasses because walls from many tissues contain little or no (1,3;1,4)-β-glucan. Secondly, there is circumstantial evidence to support a metabolic link between (1,3;1,4)-β-glucan and starch synthesis. Comparison of grain composition in a range of barley lines, including starch mutants, suggested that there is an inverse relationship between starch and (1,3;1,4)-β-glucan content in mature barley grain (Trafford and Fincher, 2014). Higher values for absolute (1,3;1,4)-β-glucan content in low-starch mutants support the notion that blocking starch synthesis can result in the diversion of glucose into (1,3;1,4)-β-glucan synthesis.

This inverse relationship between starch and (1,3;1,4)-β-glucan contents is also observed at the interspecies level. For example, (1,3;1,4)-β-glucans are the major long-term storage form of carbohydrate in the starchy endosperm of *Brachypodium distachyon* grain, which contains up to 45% (1,3;1,4)-β-glucan and only 6% starch (Guillon et al., 2011). This can be compared with the grains of cereals and most wild grasses, which have 30–70% starch as their major storage carbohydrate and generally less than 6% (1,3;1,4)-β-glucan (Trafford et al., 2013). The lower starch content of the *Brachypodium distachyon* grain is associated with much lower activities of ADP-glucose pyrophosphorylase and starch synthase in the developing grain (Trafford et al., 2013).

In a related observation, Roulin et al. (2002) showed that when young barley plants growing in the light were moved into dark conditions, the levels of (1,3;1,4)-β-glucan in their leaves dropped to close to zero but increased back to normal levels when the plants were returned to light conditions. This work led to the proposal that (1,3;1,4)-β-glucans might act as a secondary source of metabolizable glucose in young barley leaves. Morrall and Briggs (1978) calculated that hydrolysis of (1,3;1,4)-β-glucan in germinated barley grains contributed 18.5% of the carbohydrate supply to the embryo, with starch providing the remainder.

In proposing that (1,3;1,4)-β-glucans might act as a secondary source of metabolizable glucose in plant leaves, Roulin et al. (2002) pointed out that glucose derived from wall (1,3;1,4)-β-glucans could be more quickly mobilized than could glucose from starch granules. The complete depolymerization of (1,3;1,4)-β-glucan to glucose requires the action of just two enzymes, namely a (1,3;1,4)-β-glucan endohydrolase and a broad specificity, exo-acting β-glucan glucohydrolase (Hrmova and Fincher, 2007). This compares with the various amylases, debranching enzymes and α-glucosidases required for starch degradation, which is also complicated by the need for transport across the amyloplast membrane and the relatively high crystallinity of the starch granule. The concentration of (1,3;1,4)-β-glucans near vascular bundles in barley leaves would be consistent with an ability to rapidly transport glucose released from leaf (1,3;1,4)-β-glucans to other parts of the plant (Burton et al., 2011).

Similarly, it can be argued that the biosynthesis of (1,3;1,4)-β-glucans might be achieved more quickly and more efficiently than the biosynthesis of starch. A single CslF, CslH, or CslJ enzyme is sufficient for the biosynthesis of (1,3;1,4)-β-glucans, while amylose and amylopectin synthesis involves multiple starch synthases and branching enzymes, again with the potential hindrance of the amyloplast membrane and the crystallinity of the granule.

Whether or not the proposed use of (1,3;1,4)-β-glucans as a short-term storage of glucose confers any competitive advantage on the grasses has not been demonstrated; it has been shown that positive selection pressure is being exerted on the *CslF7* gene and on specific amino acid residues in the CslF6 enzyme (Schwerdt et al., 2015), as mentioned above.

## Enzymic Mechanism of (1,3;1,4)-β-Glucan Synthesis

**Figure 4:**
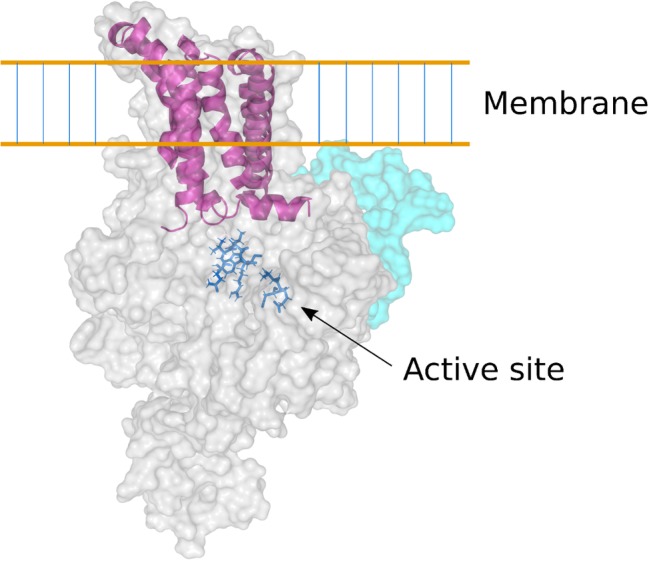
Homology model of the barley CslF6 enzyme. Based on the 3D crystal structure of a bacterial cellulose synthase enzyme (Morgan et al., 2013), this model of the barley CslF6 (1,3;1,4)-β-glucan synthase shows the TED and QVRW active site residues in dark blue stick representation. The nascent (1,3;1,4)-β-glucan chain is extruded through a membrane pore that is formed in the center of six transmembrane α-helices (shown in purple). The pale cyan region of the enzyme shows the modeled position of the insert of approximately 55 amino acid residues that is found only in the CslF6 enzyme. There is some debate as to whether the enzyme is embedded in the plasma membrane or in the Golgi membrane (Kim et al., 2015).

The complete amino acid sequences deduced from the genes encoding the (1,3;1,4)-β-glucan synthases CslF, CslH, and CslJ, coupled with the solution of the three-dimensional (3D) structure of a cellulose synthase enzyme (Morgan et al., 2013), allowed a homology model of the barley HvCslF6 enzyme to be constructed (Figure 4; Schwerdt et al., 2015). The *HvCslF6* gene appears to be the most important for mediating (1,3;1,4)-β-glucan synthesis in the developing barley endosperm (Burton et al., 2008). Its encoded membrane-bound enzyme is predicted to consist of an intracellular or intraorganellar active site from which the nascent polysaccharide is extruded to the opposite side of the membrane through a pore that is formed from six transmembrane α-helices (Figure 4). The barley CslF6 enzyme is distinguished from the other nine putative CslF enzymes in barley by the insertion of an approximately 55-amino acid residue section, which influences the amount and fine structure of the newly synthesized (1,3;1,4)-β-glucan (Schreiber et al., 2014). It is still not known if the enzyme is capable of inserting both (1,3)-β-glucosyl and (1,4)-β-glucosyl residues into the elongating polysaccharide chain or if additional enzymes are required for the insertion of the (1,3)-β-glucosyl residues (Burton et al., 2010; Kim et al., 2015). At this stage, it appears likely that the enzyme can synthesize both linkage types.

**Figure 5:**
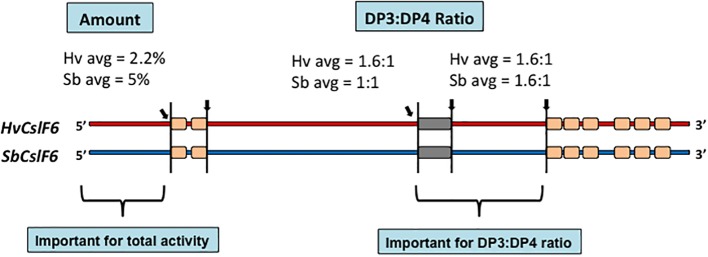
Chimeric constructs of the sorghum and barley CslF6 enzymes. When the HvCslF6 NH_2_-terminal section was replaced with the homologous region of the SbCslF6 enzyme, the amount of (1,3;1,4)-β-glucan synthesized increased in the heterologous expression system, while interchange of the sections indicated closer to the COOH-terminus changed the DP3:DP4 ratios (Dimitroff et al., 2016).

The availability of the amino acid sequence of the CslF6 enzyme enabled the identification of sections of the enzyme that are important for overall (1,3;1,4)-β-glucan synthesis and for defining the DP3:DP4 ratio of the nascent polysaccharide chain (Dimitroff et al., 2016). For this study, *CslF6* genes from *Sorghum bicolor* and *Hordeum vulgare* were expressed heterologously in *Nicotiana benthamiana.* The *SbCslF6* and *HvCslF6* genes were chosen because of the large differences in the amount of (1,3;1,4)-β-glucan synthesized in this system and in the differences in DP3:DP4 ratios of the nascent polysaccharide. The amounts of (1,3;1,4)-β-glucan synthesized were about 5 and 2.2% of grain weight, respectively, while the DP3:DP4 ratios were 1.1:1 and 1.6:1, respectively. Chimeric cDNA constructs with interchanged sections of the barley and sorghum *CslF6* genes were constructed to identify regions of the synthase enzyme responsible for these differences. The domain swapping experiments enabled the identification of regions of the enzyme important for both the total activity and for the DP3:DP4 ratio (Figure 5; Dimitroff et al., 2016). Changes of specific amino acid residues within the catalytic region of the enzymes resulted in the identification of a single G638D polymorphism upstream of the TED motif that dramatically changed the fine structure of the polysaccharide produced (Dimitroff et al., 2016). Despite these advances, it is likely that a complete understanding of the catalytic mechanism of (1,3;1,4)-β-glucan synthases will await the precise definition of the 3D structure of the enzyme.

## Increases in Heteroxylans in the Walls of Grasses

As noted above, one of the distinguishing features of the cell walls of grasses is the increase in heteroxylan content compared with walls from eudicots (Table 1). The heteroxylans of the grasses consist of a backbone of (1,4)-linked β-D-xylopyranosyl (Xyl*p*) residues, which are substituted mostly with single α-L-arabinofuranosyl (Ara*f*) or single α-D-glucuronopyranosyl (Glc*p*A) residues, or the 4-O-methyl ethers of the Glc*p*A residues (Izydorczyk and Biliaderis, 1994; Fincher and Stone, 2004). Less commonly, oligosaccharides such as β-D-Xyl*p*-(1,2)-L-Ara*f*-(1- and β-D-Gal*p*(1,4)-β-D-Xyl*p*-(1,2)-L-Ara*f*-(1- are appended to the main chain (1,4)-β-xylan (Fincher and Stone, 2004). The Ara*f* residues are mostly linked to the C(O)3 position of the Xyl*p* residues but in some cases are found on C(O)2 or on both C(O)2 and C(O)3. The Glc*p*A residues are usually linked to the C(O)2 atom of the Xyl*p* residues.

**Figure 6:**
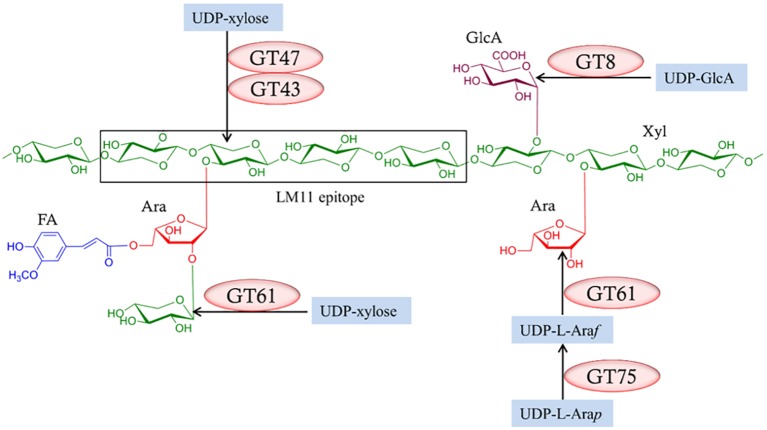
Enzymes required for the biosynthesis of heteroxylans. Several enzymes from different glycosyl transferase (GT) classes (Lombard et al., 2014; http://www.cazy.org/) are required and these might vary between species. Reproduced from Chowdhury et al. (2017).

It follows from the complexity of heteroxylan structures that their biosynthesis requires the concerted action of many enzymes (Figure 6; Chowdhury et al., 2017). Common approaches to the identification of participating genes have been to overexpress or knock down expression of candidate genes in heterologous expression systems or to examine mutant lines. Much of the work has been performed in *Arabidopsis*. Three major experimental constraints have compromised the interpretation of these approaches. Firstly, most of the plants used in plant heterologous expression systems have background levels of heteroxylans in their walls, so a clear-cut interpretation of the results is not always possible. Is the observed increase or decrease in the polysaccharide or substituent of interest truly attributable to the transgene or to some unknown pleiotropic effect? A clearer conclusion would be possible if the plant in which the gene of interest is expressed contains no heteroxylan, as shown when the *CslF* genes that encode (1,3;1,4)-β-glucan synthases were expressed in *Arabidopsis*, which contains no *CslF* genes and no (1,3;1,4)-β-glucan in its walls (Burton et al., 2006). There are several nonplant heterologous expression systems that might be used, but many of these will not contain the biochemical machinery for the synthesis of precursors such as UDP-xylose, or other crucial components needed for the overall heteroxylan structure. Secondly, many of the synthase enzymes are encoded by multiple genes and attempts to target the over- or under-expression of one gene of the family might not target the key gene family member involved in the particular tissue at the time. Furthermore, changes induced in the levels of mRNA for one gene might be compensated for by changes in transcriptional activity of other genes in the family. Thirdly, difficulties in developing reliable biochemical assays to measure heteroxylan synthase activity in microsomal preparations from mutant or transgenic lines, together with difficulties in demonstrating that the products of the enzyme assays are indeed polysaccharides of a reasonable length, have limited potential “proof-of-function” testing.

Nevertheless, these approaches have been used to identify genes that mediate heteroxylan biosynthesis, in particular genes that encode enzymes responsible for the substitution of the backbone (1,4)-β-xylan chain. Thus, family GT43, GT47, and GT61 enzymes have been implicated in the addition of Xyl*p* residues to the (1,4)-β-xylan chain, while GT8 enzymes are believed to add Glc*p*A residues to the backbone and GT61 enzymes are attributed to the addition of Ara*f* residues (Figure 6). The central question in heteroxylan biosynthesis relates to the enzymes that are required for the synthesis of the (1,4)-β-xylan backbone of the polysaccharide. Jensen et al. (2014) and Urbanowicz et al. (2014) expressed the *irregular xylem* (*IRX10*) gene in various heterologous systems and showed that the enzyme is capable of adding several xylosyl residues to oligoxyloside acceptors. Incubation times of 10–96 h were used and, where they can be calculated, the catalytic rate constants (k_cat_) appear to be quite low.

While we believe that the evidence that the IRX10 enzymes, which are members of the GT47 family, can indeed add Xyl*p* residues from UDP-xyl to oligoxylosides is strong, the apparently slow rate of addition of these xylosyl residues is not what might be expected of polysaccharide synthase enzymes, many of which have a processive action pattern. The slow rate could be explained by suboptimal conditions of *in vitro* assays. We also believe that certain unidentified members of the processive GT2 (Lombard et al., 2014)^1^ family of enzymes could mediate (1,4)-β-xylan biosynthesis and that this possibility should not be discounted at this stage. Members of the *CesA/Csl* gene family are predominantly involved in the synthesis of cell wall polysaccharides with (1,4)-β-linkages, such as cellulose, mannans/glucomannans, xyloglucans, and (1,3;1,4)-β-glucans. One wonders if the large *CesA/Csl* gene family also contains genes that mediate the iterative biosynthesis of (1,4)-β-xylans, given the relatively minor structural differences between xylosyl, glucosyl, and mannosyl residues. Little et al. (2019) recently showed that two members of the *CslF* gene group (*CslF3* and *CslF10*) were involved in the synthesis of a previously unknown glucoxylan, in which both (1,4)-β-glucosyl and (1,4)-β-xylosyl residues are present at ratios ranging from 1.5:1 to 5:1 in an unbranched polysaccharide chain. Glucosyl residues appear to predominate, so the polysaccharide might be more correctly referred to as a (1,4)-β-xyloglucan. Related polysaccharides are found in the cell walls of the marine alga, *Ulva*. The *CslF3* and *CslF10* genes form a relatively recently evolved monophyletic subgroup of the *CslF* clade (Little et al., 2019). This finding adds evidence to earlier work that all members of a single *CesA/Csl* gene clade might not necessarily be involved in the synthesis of a single polysaccharide class and has prompted us to question whether processive (1,4)-β-xylan synthases might also be present in the large GT2 *CesA/Csl* gene family.

At the broader level, it is not easy to identify factors that might have led to the increases in heteroxylans in the walls of grasses, which accompanied decreases in pectic polysaccharides and xyloglucans in these walls. The higher heteroxylan content of the walls from grasses might result passively (reactively) from the downregulation of pectic and xyloglucan biosynthesis or actively from upregulation of heteroxylan synthesis. In either case, transcription factors that influence expression patterns of multiple genes are likely to be involved. Zhang et al. (2016) profiled transcripts during the early stages of barley grain development and used spatial molecular network and gene ontology enrichment analyses to define the genes involved in cell wall biosynthesis and degradation. In addition, a co-expression network was generated using a set of transcription factors in combination with the differential subset of “cell wall” genes. The co-expression network highlighted transcription factors that are associated with specific stages of cell wall metabolism during endosperm development, and these could be considered candidate genes for the regulation of the control of heteroxylan biosynthesis.

## Co-evolution of (1,3;1,4)-β-Glucan Synthases and Hydrolases

When (1,3;1,4)-β-glucans evolved in vascular plant cell walls, it would have been energetically expensive had not a system existed for the recovery of the glucose during growth and development of the plant. In the first instance, it is likely that preexisting endo-acting cellulases could perform the depolymerization of the (1,3;1,4)-β-glucans. These enzymes are (1,4)-β-glucan endohydrolases and are widespread in plants. Endo-cellulases are encoded by relatively large gene families in the grasses, where barley, maize (*Zea mays*), sorghum (*Sorghum bicolor*), rice (*Oryza sativa*), and *Brachypodium distachyon* have from 23 to 29 genes that are expressed in a wide range of tissues (Buchanan et al., 2012). These endo-cellulases can hydrolyse (1,4)-β-glucosyl linkages in (1,3;1,4)-β-glucans, at the positions indicated below by the arrows:

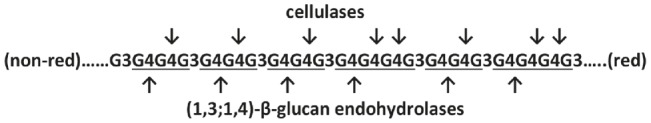

The oligosaccharides released by cellulases can be separated by HPLC and give characteristic elution patterns that differ from those released by the (1,3;1,4)-β-glucan endohydrolases. If cellulases did indeed act to hydrolyse (1,3;1,4)-β-glucans following the initial evolution of these polysaccharides, it is also likely that, in due course, enzymes that were specific for the hydrolysis of (1,3;1,4)-β-glucans would evolve, to enable more specific metabolism and regulation of these new polysaccharides. The grasses have certainly evolved specific (1,3;1,4)-β-glucan endohydrolases, which hydrolyse the polysaccharide at different positions, as indicated in the diagram above. The specificity of the (1,3;1,4)-β-glucan endohydrolases lies in the fact that they hydrolyse only (1,4)-β-glucosyl linkages and only if those linkages are immediately adjacent, on the reducing end side, to (1,3)-β-glucosyl linkages. Cellulases will not hydrolyse (1,4)-β-glucosyl linkages in this position (Anderson and Stone, 1975).

**Figure 7:**
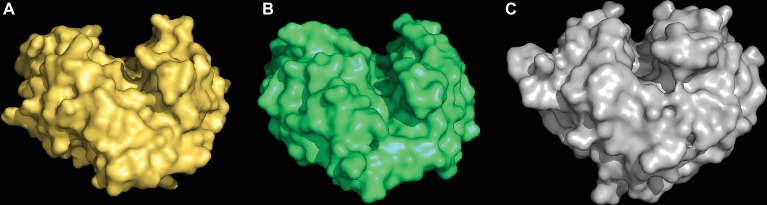
3D structures of a (1,3;1,4)-β-glucan endohydrolase **(A)**, a (1,3)-β-glucan endohydrolase **(B)** from barley, and a (1,3)-β-glucan endohydrolase **(C)** from *Bacillus subtilis*. The (1,3;1,4)-β-glucan substrate-binding clefts extend across the surface of the enzymes in each case and the catalytic acid and base residues are located within the clefts (not shown). These models were built from 3D crystal structure coordinates determined by Varghese et al. (1994) and Santos et al. (2011).

The first clue about the evolutionary origin of the specific (1,3;1,4)-β-glucan endohydrolases came from comparisons of the 3D crystal structures of a barley (1,3;1,4)-β-glucan endohydrolase and a barley (1,3)-β-glucan endohydrolase (Varghese et al., 1994). The overall amino acid sequence identity between the two enzymes was about 50%, but the C^α^ backbones of the two proteins were superimposable, with an rms deviation of 0.65 Å over 278 of the 306 residues. Both enzymes were α/β-barrels characterized by the presence of deep, substrate-binding grooves that extend across their surfaces and amino acid residues that are candidates for changing their substrate specificities were identified (Figure 7; Varghese et al., 1994).

The clear conclusion from the close similarity of these 3D structures was that the evolution of the new (1,3;1,4)-β-glucan endohydrolases arose through mutations in the more widely distributed and more ancient (1,3)-β-glucan endohydrolases. Thus, the evolution (1,3;1,4)-β-glucan endohydrolases, which hydrolyse (1,4)-β-glucosyl linkages, recruited (1,3)-β-glucan endohydrolases, which hydrolyse (1,3)-β-glucosyl linkages. The (1,3)-β-glucan endohydrolases are responsible for the mobilization of callose and other (1,3)-β-glucans in plants, but they are also members of the “pathogenesis-related” group of protective proteins, which can hydrolyse the (1,3)- and (1,3;1,6)-β-glucans in the walls of invading fungi. Both of these plant (1,3)- and (1,3;1,4)-β-glucan endohydrolases are members of the GH17 family of glycosyl hydrolases (Lombard et al., 2014)^2^. In addition, a structurally similar family GH16 (1,3;1,4)-β-glucan endohydrolase from the saprophytic bacterium *Bacillus subtilis* (strain 168) (Santos et al., 2011) exhibits the same substrate specificity as the plant enzymes and has clearly arisen through convergent evolution (Figure 7).

## Concluding Comments

The evolution of increased levels of heteroxylans in the grasses might have followed “passively” from the reduction of xyloglucan and pectin biosynthesis or “actively” through a major upregulation of xylan synthase genes or both. While the detailed mechanisms of heteroxylan synthesis and the full complement of genes involved appear to be incompletely understood at this stage, significant progress has been made in recent years and transcription data are providing candidate genes for the regulation of this process. In the case of (1,3;1,4)-β-glucan biosynthesis, the multiple routes of independent and convergent evolution of the CslF, CslH, and CslJ enzymes that mediate the process have now been defined. The *CslF* genes have been identified not only in the graminids of the Poales (which contain the Poaceae family) but also in the restiids (Figure 2). In contrast, the *CslH* and *CslJ* genes co-evolved along different pathways and have been detected in species in both the commelinid and noncommelinid monocots. However, the presence of (1,3;1,4)-β-glucan in species carrying these genes has only been confirmed through biochemical assays and/or immunocytochemistry in a small number of cases (Figure 2). Nevertheless, it is clear that the (1,3;1,4)-β-glucans are not restricted to the Poaceae in angiosperms. Although the evolution of (1,3;1,4)-β-glucan synthase activity has been investigated and important amino acid residues and domains have been identified, definition of the precise molecular mechanism through which (1,3)-β-glucosyl and (1,4)-β-glucosyl residues are added to the elongating polysaccharide chain awaits the availability of a high resolution 3D structure of the enzyme. Finally, it is also apparent that as the various (1,3;1,4)-β-glucan synthase genes and enzymes evolved, specific (1,3;1,4)-β-glucan endohydrolase enzymes co-evolved to allow the specific depolymerization of this new polysaccharide. The (1,3;1,4)-β-glucan endohydrolases appear to have evolved from the widely distributed (1,3)-β-glucan endohydrolases.

## Author Contributions

GF was invited to submit this review. VB and GF prepared the manuscript. JS prepared some of the figures and contributed to the manuscript.

### Conflict of Interest Statement

The authors declare that the research was conducted in the absence of any commercial or financial relationships that could be construed as a potential conflict of interest.

## Footnotes

^1^http://www.cazy.org/

^2^http://www.cazy.org/
